# Anxiety and Depressive Symptoms Before and During the COVID‐19 Pandemic: A Longitudinal Network Analysis

**DOI:** 10.1155/da/9620883

**Published:** 2026-03-06

**Authors:** Sophie J. Blackmore, Marit Sijbrandij, Jose Luis Ayuso-Mateos, Richard Bryant, Matteo Monzio Compagnoni, Katalin Gémes, Erik J. Giltay, Almar A. L. Kok, Vincent Lorant, Roberto Mediavilla, Josep Maria Haro, Raffael Kalisch, Maria Melchior, Ellenor Mittendorfer-Rutz, Pablo Nicaise, Papoula Petri-Romão, Judith van der Waerden, Henrik Walter, Anke B. Witteveen, Brenda W. J. H. Penninx

**Affiliations:** ^1^ Department of Clinical, Neuro- and Developmental Psychology, Vrije Universiteit Amsterdam, Amsterdam, Netherlands, vu.nl; ^2^ Department of Education and Counselling Psychology, McGill University, 3700 McTavish Street, Montreal, Quebec, H3A 1Y2, Canada, mcgill.ca; ^3^ Department of Clinical, Neuro- and Developmental Psychology, WHO Collaborating Center for Research and Dissemination of Psychological Interventions, Amsterdam Public Health Research Institute, Vrije Universiteit Amsterdam, Amsterdam, Netherlands, vu.nl; ^4^ Department of Psychiatry, Instituto de Investigación Sanitaria - Hospital Universitario La Princesa, Madrid, Spain, uam.es; ^5^ Centro de Investigación Biomédica en Red de Salud Mental, Instituto de Salud Carlos III, Madrid, Spain, isciii.es; ^6^ Department of Psychiatry, Universidad Autónoma de Madrid, Madrid, Spain, uam.es; ^7^ School of Psychology, UNSW Sydney, Sydney, Australia, unsw.edu.au; ^8^ National Centre for Healthcare Research and Pharmacoepidemiology, University of Milano-Bicocca, Milan, Italy, unimib.it; ^9^ Department of Statistics and Quantitative Methods, Unit of Biostatistics, Epidemiology and Public Health, University of Milano-Bicocca, Milan, Italy, unimib.it; ^10^ Division of Insurance Medicine, Department of Clinical Neuroscience, Karolinska Institutet, Stockholm, Sweden, ki.se; ^11^ Department of Psychiatry, Leiden University Medical Center, Leiden, Netherlands, lumc.nl; ^12^ Health Campus the Hague - Leiden University, The Hague, Netherlands, lumc.nl; ^13^ Department of Psychiatry, Amsterdam UMC Location Vrije Universiteit Amsterdam, Mental Health Programme, Amsterdam Public Health, Amsterdam, Netherlands, vu.nl; ^14^ Institute of Health and Society (IRSS), Université Catholique de Louvain (UCLouvain), Brussels, Belgium, uclouvain.be; ^15^ Research and Development Unit, Parc Sanitari Sant Joan de Déu, Barcelona, 08830, Spain, pssjd.org; ^16^ Leibniz Institute for Resilience Research, Mainz, Germany; ^17^ Neuroimaging Center (NIC), Focus Program Translational Neuroscience (FTN), Johannes Gutenberg University Medical Center, Mainz, Germany, unimedizin-mainz.de; ^18^ INSERM, Institut Pierre Louis d’Épidémiologie et de Santé Publique, IPLESP, Epidémiologie Sociale, Santé Mentale et Addictions ESSMA, Sorbonne Université, Paris, 75012, France, sorbonne-universites.fr; ^19^ Department of Psychiatry and Psychotherapy, Charité, Universitätsmedizin, Berlin, Germany; ^20^ Department of Psychiatry, Amsterdam University Medical Center, Amsterdam Public Health, Vrije Universiteit, Amsterdam, Netherlands, vu.nl

## Abstract

**Background:**

Despite the societal impact of the COVID‐19 pandemic, research assessing changes in symptoms of anxiety and depression following the sanitary crisis has reported heterogeneous evidence, especially among individuals with pre‐existing mental health conditions. Most earlier studies used summary scores of depression or anxiety assessment surveys, which does not provide insights into changes in individual symptoms and symptom structure during the pandemic.

**Objective:**

This study used a network analysis to investigate the symptom structure of anxiety and depressive symptoms and temporal changes in symptoms across three timepoints before and during the COVID‐19 pandemic in a sample primarily of persons with anxiety and depressive disorders.

**Methods:**

Data are retrieved from 675 participants of the Netherlands Study of Depression and Anxiety (NESDA) and Netherlands Study of Depression in Older Persons (NESDO) cohorts who completed all three timepoints. Anxiety and depressive symptoms were assessed using the Beck anxiety inventory (BAI) and the Quick Inventory of Depressive Symptomatology–Self‐Rated (QIDS–SR). Symptom networks were estimated for three timepoints: pre‐COVID, first COVID‐peak (April 2020), second COVID‐peak (January/February 2021), and compared. Symptom importance was quantified using centrality indices (strength, betweenness, and closeness), and temporal stability was assessed using the network comparison test (NCT).

**Results:**

The symptom networks were largely consistent between the two COVID‐19 peak timepoints but differed significantly from the pre‐COVID network in terms of global strength. Fear‐related anxiety symptoms, sleep‐related symptoms, and thoughts of death or suicide demonstrated greater strength centrality at the pre‐COVID timepoint compared to both COVID‐19 peak timepoints.

**Conclusion:**

Our findings illustrate that a stressful situation, such as the COVID‐19 pandemic, can result in changes in the complex associations of anxiety and depressive symptoms. Unexpectedly, anxiety symptom networks shifted from being driven by somatic fear responses before the pandemic, toward greater prominence of cognitive appraisal symptoms during the pandemic. Network insights could help focus intervention planning by targeting the key symptoms of anxiety and depression.

## 1. Introduction

The World Health Organization declared COVID‐19 a global pandemic on March 11, 2020 [1]. Across the world, safety measures were implemented to mitigate the impact of the crisis [2]. While these measures were essential in stopping the spread of COVID‐19, they also significantly impacted the daily lives of all citizens. In previous health crises, these practices, specifically isolating or quarantining away from others, as well as stress and fear caused by these events, have been linked to increased reports of psychological distress, including post‐traumatic stress symptoms, emotional exhaustion, depression/low mood, and irritability [3].

Longitudinal studies assessing the mental health impact of the crisis have found heterogeneous evidence of changes in anxiety and depression symptoms during the pandemic. While an umbrella review found that pooled longitudinal data consistently demonstrated a small increase in depression and anxiety symptoms within the general population throughout the pandemic, no increase was found for people with pre‐existing mental disorders [4]. A systematic review and meta‐analysis including only primary studies with pre‐pandemic comparisons found no or only small changes in anxiety or depression symptoms among the general population compared to pre‐pandemic levels [5]. Regarding individuals with diagnosed mental disorders, this meta‐analysis found a small, but significant improvement in general mental health and depression symptoms for people with pre‐existing diagnoses, however these findings were based on a small number of studies [5]. Indeed, in our analyses of three Dutch cohort studies [6], we also found that while individuals with psychiatric diagnoses scored higher on all symptom scales (depression, anxiety, worry, and loneliness) during the pandemic, they did not report a greater increase in any of the symptoms as compared to pre‐pandemic times. There were even decreases in depression and worry symptoms observed in those with the most severe mental health disorder burden [6].

Anxiety and depressive symptoms represent key aspects of mental health that are sensitive to both chronic vulnerabilities and acute environmental stressors. Before the COVID‐19 pandemic, these symptoms often reflected ongoing psychiatric burden, whereas during the pandemic they may also have captured reactions to stressors such as fear of infection, uncertainty, social isolation, and changes in daily structure. Understanding these symptoms in both periods is important for clarifying whether the pandemic altered their expression and interconnections. One reason why some studies found no change or symptom improvements throughout the pandemic among individuals diagnosed with a psychiatric disorder may be that studies tend to evaluate depression and anxiety symptoms using total scores of standard scales [7]. However, both depression and anxiety comprise a cluster of symptoms, and by combining them into a composite score and sum‐scores, the ability to assess associations and inter‐relationships between individual symptoms is lost [7]. It is possible that throughout the pandemic some of the individual anxiety and depression symptoms increased (e.g., distress), while others decreased (e.g., sleep dysregulations) [8]. For example, while the ability to work from home and decreased social obligations may have provided some relief from anxiety symptoms, mandated quarantine measures and social isolation might exacerbate depression symptoms [9]. This information, when presented as a sum score, may obscure more subtle specific symptom change that could have occurred. Therefore, we explored anxiety and depressive symptoms at the symptom level rather than at the total‐score level, to capture how specific aspects of distress shifted across time. Investigating these patterns helps clarify which symptoms are most reactive to the pandemic context, and why some individuals with pre‐existing mental disorders showed stability or even improvements despite widespread societal stress.

Network analysis, based on the theoretical network model, models interactions between symptoms, positing that psychological attributes are complex systems of observable behaviors that mutually and dynamically reinforce one another [10]. Network analysis also allows the identification of “central” symptoms (symptoms that are strongly connected with other symptoms) [7]. The identification of these central symptoms which activate other symptoms in a network is important as these may represent key targets in treatment or intervention planning.

Network analysis has been used to assess various aspects of the COVID‐19 pandemic, including disease transmission and efficacy of quarantine measures [11, 12], the economic impact of the pandemic [13], and the impact of COVID‐19 on mental health symptoms in various populations (i.e., [14–16]). Most of these studies, however, did not include a pre‐pandemic assessment and were, therefore, unable to assess changes in network structure over time. One of the few longitudinal network analyses was carried out in the UK with data from before (2019), at the onset of (April 2020), and during (November 2020) the COVID‐19 pandemic [16]. They found rather consistent patterns of symptom relationships over time, and that loneliness and worthlessness emerged as the most central symptoms across their networks [16]. Another study compared two cross‐sectional networks of different samples of participants with depression and anxiety before and during COVID‐19 in outpatient populations in Korea and observed a structural change between the two networks and that, while feeling of guilt was the most central symptom in the network pre‐pandemic, somatic anxiety emerged as the most central symptom during the pandemic [17].

The current study used network analysis to investigate symptom structure and temporal stability of anxiety and depressive symptoms at three timepoints before and during the COVID‐19 pandemic in a cohort primarily of persons with diagnosed mental health disorders in the Netherlands. Specifically, we aimed to (1) identify three networks corresponding to a baseline, pre‐pandemic timepoint, and two timepoints corresponding to the periods of highest COVID‐19 restrictions in the Netherlands; (2) interpret the importance of symptoms in each network by using the centrality measures of betweenness, closeness, and strength; and (3) assess changes in network structure, network strength, and centrality of symptoms between baseline and COVID‐19 timepoints using the network comparison test (NCT). We hypothesized that network structure would vary significantly pre‐ and during‐pandemic, with greater similarity in strength and network structure between the two during‐COVID timepoints vs. between the baseline and during‐COVID timepoints. We also hypothesized that the centrality of certain symptoms will vary significantly across the timepoints. First, we expected to find a greater centrality of fear‐related anxiety symptoms at the during‐COVID timepoints, based on the novel stress brought on by the pandemic, its high rate of transmission and infection, and associated isolation measures. Finally, due to more flexible work from home arrangements and less activity, we expected that people had greater control/ability to manage their sleep, and; therefore, hypothesized that sleep symptoms will have significantly lower centrality in the two during‐COVID timepoints compared to the baseline.

## 2. Methods

### 2.1. Participants

This study used data from participants recruited in two cohort studies conducted in the Netherlands: the Netherlands Study of Depression and Anxiety (NESDA) and the Netherlands Study of Depression in Older Persons (NESDO). NESDA is a longitudinal study assessing the development and course of depression and anxiety among people aged 18–65 years with a depression or anxiety disorder (*n* = 2329), biological siblings (*n* = 367), and individuals without a lifetime mental health disorder (*n* = 652). Participants were recruited from community, primary and specialized mental health care in the Netherlands between 2004 and 2007 and followed up after 2, 4, 6, and 9 years, and at 16 timepoints throughout the COVID‐19 pandemic.

NESDO is a longitudinal study examining the development and course of depression in a cohort of older patients aged 60–93 years with a depressive disorder (*n* = 378) or without lifetime mental health disorders (*n* = 132). Participants were recruited from primary care from 2007 to 2010 and followed up after 2 and 6 years, and at 16 timepoints throughout the COVID‐19 pandemic. For more information of the NESDA/NESDO samples, please see Pan et al. [6].

The COVID‐19 study was approved by the Institutional Review Board of Vrije Universiteit Medical Center, Amsterdam (Reference Number 2020.166), and adhered to the Declaration of Helsinki. All participants provided informed consent online.

Three timepoints were used for network analysis in this study: a pre‐COVID timepoint (T0), and two timepoints collected during the COVID‐19 pandemic (T1 and T2). Data for the pre‐COVID timepoint was gathered from the most recent NESDA and NESDO follow‐up data collection available pre‐pandemic, which was collected from 2013 to 2016 (NESDA: 9‐year follow‐up; NESDO: 6‐year follow‐up). The two timepoints during‐COVID were selected to represent the times of highest restriction measures in the Netherlands, indicated by the COVID‐19 stringency index [18]. These timepoints were April 2020 (Wave 2 and 3 of original COVID data collection, i.e., W2_NESDA/NESDO_ and W3_NESDA/NESDO_) and January/February 2021 (Wave 11 and 12 of original COVID data collection, i.e., W11_NESDA/NESDO_ and W11_NESDA/NESDO_). For the two COVID timepoints, participants who completed one or both waves (W2 and/or W3; W11 and/or W12) were included, whereby when two waves of information per timepoint was present this was averaged.

Participants were only included if they had completed the baseline timepoint and the two COVID timepoints, resulting in a final sample of 675 participants.

### 2.2. Measures

#### 2.2.1. Sociodemographic variables

Information on age, gender, and education level (years attained) was gathered from the pre‐COVID data collection timepoint. This information was not collected during the COVID timepoints.

Depression and anxiety scores for all participants were gathered from each timepoint.

#### 2.2.2. Beck Anxiety Inventory (BAI)

The BAI is a 21‐item self‐report questionnaire that measures somatic and cognitive symptoms of anxiety in adolescents and adults (BAI; [19]; Dutch translation; [20]). Items are rated on a Likert scale ranging from 0 (not at all) to 3 (it bothered me a lot), with a maximum score of 63 and BAI scores are classified as follows: minimal level of anxiety (0–7); mild anxiety (8–15); moderate anxiety (16–25); and severe anxiety (26–63; [19]).

#### 2.2.3. Quick Inventory of Depressive Symptomatology–Self‐Rated, 16‐item (QIDS–SR‐16)

The QIDS–SR‐16 is a 16‐item self‐report measure derived from the 30‐item inventory of depressive symptomatology (IDS) designed to assess the severity of depressive symptoms [21]. Items on the QIDS correlate with the nine DSM‐IV symptom criterion domains for depression, and each item is rated on a scale of 0–3, with higher scores indicating greater symptom severity, for a maximum score of 27. Scores are classified as follows: no depression (0–5); mild depression (6–10); moderate depression (11–15); severe depression (16–20); and very severe depression (21–27) [21]. During data collection, the variables assessing appetite increase/decrease (QIDS 6 and QIDS 7) and weight increase/decrease (QIDS 8 and QIDS 9) were each combined to create one item measuring appetite change and one measuring weight change.

### 2.3. Analysis

#### 2.3.1. Demographics

Sample characteristics were calculated in SPSS. *T*‐tests (for continuous variables) and chi‐squared tests (for categorical variables) were conducted to measure differences between the included and excluded samples at the baseline timepoint.

#### 2.3.2. Network Estimation

Psychometric network analysis uses a simple visual representation in which nodes represent questionnaire items/symptoms, and the edges between them are operationalized as the partial correlation coefficient between two symptoms, or nodes [22]. The darker and wider the edge represented in the network, the stronger the correlation coefficient between two nodes. This current study estimated and visualized three networks using the qgraph and bootnet R‐packages: pre‐COVID baseline (T0), COVID timepoint 1 (T1; April 2020), and COVID timepoint 2 (T2; January 2021; [10, 23]). Prior to estimation, all symptom variables were transformed using a nonparanormal transformation to better approximate multivariate normality and address the ordinal scaling of items. The three networks were then estimated via Gaussian graphical models (GGMs) and regularized with the graphical least shrinkage and selection operator (gLASSO) to provide a more parsimonious network by eliminating insignificant or very small edge values from the network [10]. Specifically, the extended Bayesian information criterion (EBIC) was used to select the best‐fitting model, setting its hyper parameter to default, or *γ* = 0.5, to ensure a more conservative network estimation [10, 24]. To evaluate the robustness of network estimation to hyperparameter choice, we also conducted a sensitivity analysis by re‐estimating networks at each timepoint using EBIC *γ* values of 0, 0.25, and 0.50. The resulting networks were compared by computing correlations of edge weights across *γ* specifications and by visually inspecting the corresponding network plots (Supporting Information 1: Appendix Figures E1–E3).

#### 2.3.3. Network Inference

To quantify the importance of nodes, three indices of centrality were calculated for each network: strength, betweenness, and closeness [25]. Strength refers to the weighted sum of all associations between a given node and all other nodes in the network and captures the total influence that a node has in the network [25]. Betweenness refers to the frequency that a certain specific node lies on the shortest path between two other nodes and indicates how efficiently a node is connected to other nodes [25]. Closeness refers to the average distance from a given node to all other nodes and informs how quickly one node may impact another node when activated [25].

The stability of centrality indices was assessed by investigating the centrality indices after dropping a subset of cases from the network (case‐dropping subset bootstrap) [10]. This is quantified using a correlation stability coefficient (CS‐coefficient) which represents the maximum proportion of cases that can be dropped, such that there remains 95% probability the correlation between original centrality indices and centrality of subset‐based networks is 0.7 or higher [10]. For interpretation, CS values of ≥0.25 are considered minimally acceptable, while values of ≥0.50 are preferable to indicate adequate stability [10].

To evaluate the precision of edge estimates, nonparametric bootstrapping was also conducted to compute 95% confidence intervals (CIs) around edge weights. Edges with CIs excluding zero were considered more robust, though interpretation emphasizes the overall network structure rather than individual edge significance [10].

#### 2.3.4. Temporal Network Stability

To assess changes between the networks across the study timepoints, the R‐package NCT was used, which implements a permutation test that assesses difference between two selected networks [26, 27]. The NCT was used to make the following comparisons among the considered timepoints: T1 vs. T0; T2 vs. T0; and T2 vs. T1. The NCT compares networks based on several factors: network invariance (comparing the networks as a whole), global strength (comparing overall connectivity between networks), and centrality invariance (testing whether the strength of a node is identical across networks) [27]. Because the same individuals contributed data at all three timepoints, the assumption of independent samples could not be made. Therefore, the permutation tests were conducted using the paired = TRUE option in the NCT‐package, ensuring that within‐person dependency was correctly modeled. The results of the NCT are expressed as a *p*‐value, which is set against an alpha level of 0.05.

## 3. Results

### 3.1. Study Sample

A total of 2369 participants (NESDA = 2070 and NESDO = 299) completed the pre‐COVID timepoint (T0), followed by 766 participants (NESDA = 715 and NESDO = 51) who completed the first COVID timepoint (T1), and 746 participants (NESDA = 702 and NESDO = 44) who completed the second COVID timepoint (T2). For this study’s analysis, a subsample of 675 participants who completed all three study timepoints was used. Of this subsample, 77.2% (*n* = 521) met criteria for at least one lifetime mental health disorder, whereas 22.8% (*n* = 154) did not. This was calculated by Pan et al. [6] and corresponded to a lifetime presence of at least one of six disorders (major depressive disorder, dysthymia, general anxiety disorder, panic disorder, social phobia, and agoraphobia) as assessed by the DSM‐IV–based composite interview diagnostic instrument at the baseline timepoint [28].

This subsample had more women (*n* = 442, 65.6%) than men (*n* = 232, 34.4%), a mean age of 54.9 (SD = 12.5) at the pre‐COVID T0 timepoint, and an average of 13.1 education years attained (SD = 3.2). The study sample had a mean BAI score of 28.3 (SD = 8.4) at pre‐COVID T0, 27.9 (SD = 7.9) at COVID T1 (April 2020), and 27.7 (SD = 8.1) at COVID T2 (January/February 2021). The subsample had a mean QIDS score of 21.9 (SD = 6.6) at pre‐COVID T0, 22.8 (SD = 6.6) at COVID T1 (April 2020), and 22.4 (SD = 6.7) at COVID T2 (January/February 2021).

Demographic characteristics for both included and excluded samples are summarized in Supporting Information 2: Appendix Table A1. Included participants (*n* = 675) as compared to excluded participants (*n* = 1693, due to missing data during the pandemic) were not different in terms of gender or BAI scores, but were slightly older, higher educated, and less depressed. For full comparison results, see Supporting Information 3: Appendix Tables B1 and B2.

### 3.2. Network Structure and Estimation

For each of the three study timepoints, networks of anxiety and depression symptoms were estimated, shown in Figure 1a–c. The pre‐COVID T0 network had 261/595 (43.9%) non‐zero edges with a mean weight of 0.0278; the COVID T1 network had 238/595 (40%) non‐zero edges with a mean weight of 0.0266; and the COVID T2 network had 191/595 (32.1%) with a mean weight of 0.0255. Across all three networks, there were strong connections (edge weights) between the following nodes: BAI 12 (“hands trembling”)—BAI 13 (“shaky, unsteady;” T0 = 0.4555; T1 = 0.5311; T2 = 0.5154); BAI 2 (“feeling hot”)—BAI 20 (‘face flushed;’ T0 = 0.4270; T1 = 0.3675; T2 = 0.4459); BAI 2 (“feeling hot”)—BAI 21 (“hot, cold sweats;” T0 = 0.3388; T1 = 0.3858; T2 = 0.3365), and IDS 11 (“change in appetite”)—IDS 12 (“weight change;” T0 = 0.2984; T1 = 0.5003; T2 = 0.3746).

Figure 1(a–c) Network estimation for pre‐COVID T0, COVID T1, and COVID T2. BAI item label abbreviations: BAI 1, “Numbness or tingling;” BAI 2, “Feeling hot;” BAI 3, “Wobbliness in legs;” BAI 4, “Unable to relax;” BAI 5, “Fear of worst happening;” BAI 6, “Dizzy or lightheaded;” BAI 7, “Heart pounding, racing;” BAI 8, “Unsteady;” BAI 9, “Terrified or afraid;” BAI 10, “Nervous;” BAI 11, “Feeling of choking;” BAI 12, “Hands trembling;” BAI 13, “Shaky, unsteady;” BAI 14, “Fear of losing control;” BAI 15, “Difficulty in breathing;” BAI 16, “Fear of dying;” BAI 17, “Scared;” BAI 18, “Indigestion;” BAI 19, “Faint, lightheaded;” BAI 20, “Face flushed;” BAI 21, “Hot, cold sweats.” IDS 1, “Falling asleep;” IDS 2, “Sleep during the night;” IDS 3, “Waking up too early;” IDS 4, “Sleeping too much;” IDS 5, “Feeling sad;” IDS 6, “Change in appetite;” IDS 7, “Weight change;” IDS 8, “Concentration/decision making;” IDS 9, “Energy level;” IDS 10, “Thoughts of death or suicide;” IDS 11, “General interest;” IDS 12, “Feeling restless;” IDS 13, “Feeling slowed down;” IDS 14, “View of myself”.

**Figure:**
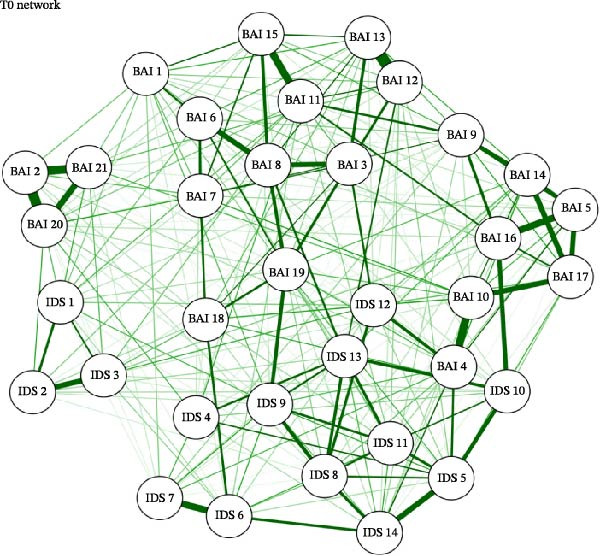
(a)

**Figure:**
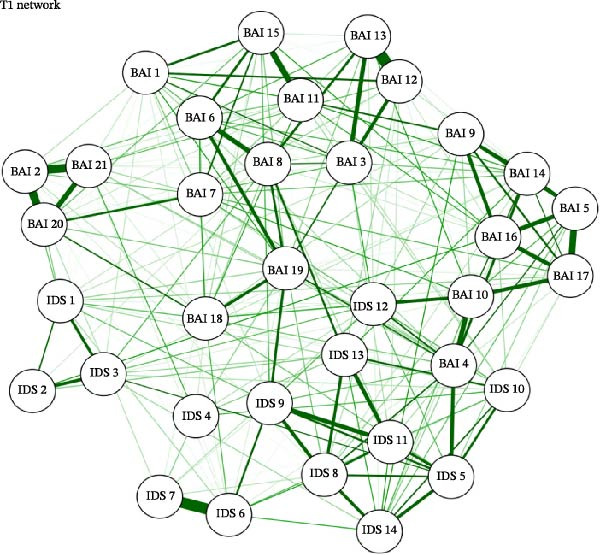
(b)

**Figure:**
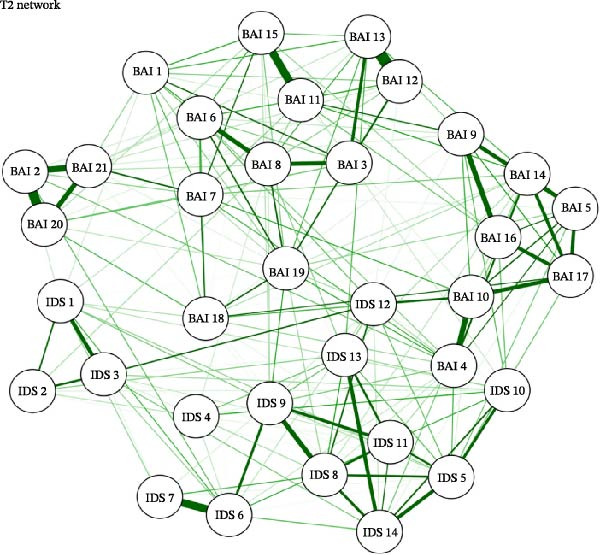
(c)

To test whether network estimation was sensitive to hyperparameter selection, we re‐estimated all three networks using EBIC *γ* values of 0, 0.25, and 0.50. Across timepoints, the resulting networks were highly consistent: edge‐weight correlations between *γ* specifications were uniformly 1.00 at T0, T1, and T2, indicating that network structure and connectivity did not vary with different *γ* choices. Sensitivity plots for each timepoint are provided in Supporting Information 1: Appendix Figures E1–E3. These findings support the robustness of the main results under the conventional *γ* = 0.50 specification.

### 3.3. Network Inference

#### 3.3.1. Pre‐COVID T0

The items with the highest betweenness centrality were BAI 8 (57; “unsteady”) and IDS 13 (64; “feeling slowed down”). The items with the highest closeness centrality were also BAI 8 (0.0016; “unsteady”) and IDS 13 (0.00164; “feeling slowed down”). The items with the highest strength centrality were BAI 13 (1.1612; “shaky/unsteady”), BAI 19 (1.1510; “faint, lightheaded”), and IDS 5 (1.1536; “feeling sad”).

#### 3.3.2. COVID T1 (April 2020)

The items with the highest betweenness centrality were BAI 19 (67; “faint, lightheaded”) and IDS 9 (79; “energy level”). The items with the highest closeness centrality were BAI 4 (0.00161; “unable to relax”) and BAI 19 (0.00174; “faint, lightheaded”). The items with the highest strength centrality were BAI 4 (1.2155; “unable to relax”), BAI 13 (1.1298; “shaky/unsteady”), and BAI 19 (1.1564; “faint, lightheaded”).

#### 3.3.3. COVID T2 (January/February 2021)

The items with the highest betweenness centrality were IDS 9 (74; “energy level”) and IDS 12 (73; “feeling restless”). The items with the highest closeness centrality were BAI 4 (0.00168; “unable to relax”) and BAI 10 (0.00165; “nervous”). The items with the highest strength centrality were BAI 4 (1.1076; “unable to relax”), BAI 13 (1.2980; “shaky, unsteady”), and IDS 8 (1.2110; “concentration/decision making”).

However, as the following stability analysis indicated that betweenness and closeness indices were not robust (CS <0.25 and <0.6, respectively), interpretations based on these measures should be made with extreme caution. The full strength, betweenness, and closeness statistics for each item at the three timepoints can be seen in Supporting Information 4: Appendix Figures C1–C3.

The robustness (accuracy and stability) of centrality indices was assessed by comparing each timepoint network to bootstrapped samples. The CS‐coefficient indicated that the centrality metric betweenness is relatively unstable across all three timepoints (CS_T0_ = 0.127; CS_T1_ = 0.283; and CS_T2_ = 0.206). Closeness performed better across the networks (CS_T0_ = 0.517; CS_T1_ = 0.594; and CS_T2_ = 0.594). Across all three timepoints, strength centrality was the most stable (CS = 0.75). As such, this study used strength centrality as the main measure to assess node importance, which is consistent with previous studies [10, 29]. For full centrality stability plots, see Supporting Information 5: Appendix Figures D1–D3.

Bootstrapped 95% CIs were computed for all estimated edges at T0, T1, and T2. Only a subset of edges demonstrated CIs excluding zero, indicating statistically reliable associations. At T0, significant connections included both within‐scale (e.g., BAI1–BAI6 and BAI12–BAI13) and cross‐scale edges (e.g., BAI10–IDS12 and BAI18–IDS6). At T1, stable associations again spanned anxiety–anxiety (e.g., BAI1–BAI12 and BAI10–BAI17), depression–depression (e.g., IDS1–IDS2 and IDS10–IDS14), and cross‐scale links (e.g., BAI4–IDS12). At T2, a similar pattern was observed, with reliable connections such as BAI1–BAI13, BAI17–IDS5, and IDS6–IDS11.

Although the majority of edges showed wide CIs overlapping zero, these subsets demonstrated relative stability across time. Full edge‐weight estimates and corresponding 95% CIs for all significant edges are reported in Supporting Informations 1–6.

### 3.4. Temporal Network Stability

The three timepoint networks were compared to each other, with results summarized in Table 1.

**Table 1:** Table summarizing results of the network comparison test.

	Pre‐COVID T0 vs. COVID T1	Pre‐COVID T0 vs. COVID T2	COVID T1 vs. COVID T2
Network invariance test			
Test statistic *p*‐Value	0.2132 0.55	0.2132 0.52	0.1508 0.98
Global strength test			
Per group Test statistic *p*‐Value	16.70, 15.20 1.4924 <0.01	16.7, 15.24 1.4581 <0.01	15.20, 15.24 0.0343 0.88
Centrality invariance test			
Item (*p*‐value)	BAI 1 (0.03)	BAI 6 (0.03) ^∗∗^	BAI 4 (0.05)
	BAI 4 (0.00) ^∗^	BAI 9 (0.01)	
	BAI 5 (0.03) ^∗^	BAI 11 (0.00)	
	BAI 9 (0.00)	BAI 18 (0.05)	
	BAI 11 (0.00)	BAI 21 (0.01)	
	IDS 1 (0.04)	IDS 2 (0.00)	
	IDS 2 (0.00)	IDS 4 (0.00)	
	IDS 4 (0.00)	IDS 9 (0.00)	
	IDS 9 (0.00)	IDS 10 (0.00)	
	IDS 10 (0.00)	IDS 12 (0.02)	
	IDS 12 (0.03)	IDS 14 (0.00) ^∗∗^	
	IDS 14 (0.00) ^∗^		

*Note:* ^∗^ = Item had significantly greater strength centrality at T1;  ^∗∗^ = Item had significantly greater strength centrality at T2; no  ^∗^ = Item had significantly greater strength centrality at baseline timepoint.

#### 3.4.1. Pre‐COVID T0 vs. COVID T1

The two networks were not significantly different regarding their invariant network structure (*M* = 0.2132, *p* = 0.55), indicating no significant differences in network structure between T0 and T1. They were significantly different regarding their global strength (*S* = 1.4924, *p* = 0.00), with the T0 network significantly more densely connected than T1. Items BAI 1 (“numbness/tingling”), BAI 9 (“terrified/afraid”), BAI 11 (“feeling of choking”), IDS 1 (“falling asleep”), IDS 2 (“sleep during the night”), IDS 4 (“sleeping too much”), IDS 10 (“thoughts of death or suicide”), and IDS 12 (“feeling restless”) had significantly greater strength centrality at the baseline timepoint compared to T1. Items BAI 4 (“not being able to relax”), BAI 5 (“fear that the worst will happen”), and IDS 14 (“view of myself”) had significantly greater strength centrality at T1 compared to T0.

#### 3.4.2. Pre‐COVID T0 vs. COVID T2

The two networks were not significantly different regarding their network structure (*M* = 0.2132, *p* = 0.52). The two networks were significantly different regarding their global strength (*S* = 1.4581, *p* = 0.00), with the T0 network significantly more densely connected than T2. The following items had significantly greater strength centrality at the pre‐COVID (T0) timepoint compared to COVID T2 (January/February 2021): BAI 9 (“terrified”), BAI 11 (“feeling of choking”), BAI 18 (“upset stomach”), BAI 21 (“hot/cold sweats”), IDS 2 (“sleep during the night”), IDS 4 (“sleeping too much”), IDS 10 (“thoughts of death or suicide”), IDS 9 (“energy level”), and IDS 12 (“feeling restless”). Items BAI 6 (“dizzy/lightheaded”) and IDS 14 (“view of myself”) both had significantly greater strength centrality at T2 than at T0.

#### 3.4.3. COVID T1 vs. COVID T2

The two networks did not differ significantly in their network structure (*M* = 0.1508, *p* = 0.98) or global strength (*S* = 0.0343, *p* = 0.88). Regarding centrality invariance testing, item BAI 4 (“not being able to relax”) had significantly greater strength centrality at COVID T1 vs. COVID T2.

## 4. Discussion

The current study is the first, to our knowledge, that used network analysis to assess anxiety and depressive symptoms in a longitudinal cohort predominantly consisting of participants with mental disorder at different timepoints throughout the COVID‐19 pandemic. Specifically, this study estimated and compared three networks, corresponding to a pre‐COVID timepoint (T0) and two timepoints during the pandemic which reflected periods of highest restriction in the Netherlands: COVID T1 (April 2020) and COVID T2 (January/February 2021).

We found that the baseline network was significantly different in global strength from the T1 and T2 timepoints, with the pre‐COVID T0 network significantly more densely connected than the during‐COVID timepoints. Network global strength refers to how well connected a network is, indicating the number of estimated edges relative to the number of edges if the network were fully connected. Some studies have suggested that more strongly connected networks will feature stronger feedback and reinforcement among symptoms, and thus lead to higher levels of illness vulnerability and less positive treatment prospects for individuals [20, 30]. Our finding that the baseline pre‐pandemic network was more densely connected than the during‐COVID timepoints might suggest that, before the pandemic, symptoms were influencing each other to some extent, however during the pandemic this influence was weaker, perhaps because the external context affected the symptomatology to a stronger degree than before the pandemic.

We also found that numerous variables differed significantly in their strength centrality between timepoints. In contrast to what we hypothesized; we did not find that acute fear symptoms of anxiety had stronger strength centrality at the COVID timepoints compared to pre‐COVID. On the contrary, many of these symptoms were more central at the pre‐COVID T0 timepoint than COVID T1 and COVID T2 respectively (i.e., “numbness/tingling;” “feeling of choking;” “upset stomach;” and “sweating”). While we had expected the pandemic to act as a fear‐inducing stressor, it is possible that symptomatology during the pandemic is not as driven by fear and distress as in nonpandemic conditions [31]. What we did find is that symptoms of anxiety more related to cognitive appraisal had higher strength centrality at the during‐COVID timepoints (i.e., “not being able to relax;” “fear that the worst will happen”…). Perhaps as people tried to adapt to constantly evolving health information and restriction measures, more appraisal‐oriented symptoms of anxiety became increasingly central within the symptom network.

We hypothesized that sleep symptoms would have lower centrality in the COVID networks compared to the pre‐COVID timepoint [8]. Indeed, we confirmed that of the four items related to sleep concerns, two items (“sleep during the night” and “sleeping too much”) decreased in strength centrality significantly between the pre‐COVID and first COVID timepoint, and then again between the first and second COVID timepoints. The other two sleep items (“falling asleep” and “waking up too early”) also decreased in strength centrality pre‐ vs. during‐pandemic, however, the decrease was nonsignificant. It is possible that while other dimensions of anxiety and depression worsened throughout the COVID‐19 pandemic, the ability to stay/work at home with diminished social responsibilities seemed associated with improved sleep conditions and lessened the importance of these nodes within the networks [8].

Interestingly, we also found that the item related to “thoughts of death or suicide” had significantly greater strength centrality in the pre‐COVID T0 vs. both during‐COVID timepoints. While one may expect increased odds of suicidal behaviors during a pandemic due to decreases in social support and difficulties adapting to new contexts, it is possible that our finding does not reflect an actual decrease in suicidal thought or risk during the pandemic but rather weakened connections between this node and others in the network during the pandemic timepoints [32]. However, in line with the current study’s findings, recent research by Pirkis et al. [33] has also found lower‐than‐expected numbers of suicides during the first year of the COVID‐19 pandemic suggesting the need for further research to clarify this relationship.

Taken together, these findings highlight the value of applying a longitudinal network approach with a minimum dataset of consistently measured symptoms. By using identical items from the BAI and QIDS across all timepoints, this study ensured comparability in symptom‐level dynamics. The longitudinal design allowed us to capture both the immediate impact of the first COVID‐19 restrictions and the more sustained effects during a later lockdown, offering insights that cross‐sectional designs cannot provide.

Clinically, the distinction between acute fear‐ and appraisal‐based anxiety symptoms has important implications. Appraisal‐based symptoms are often tied to a chronic over‐arousal for dealing with perceived dangers which is reflected through heightened attentional biases for threat and sustained apprehension which both reinforce one’s anxious thoughts [34]. While mindfulness or relaxation‐based interventions were widely used during the pandemic, our findings suggest that interventions targeting maladaptive cognitive appraisal (e.g., CBT or cognitive reappraisal strategies) may be more effective in addressing anxiety and depressive symptom dynamics. The reduced centrality of sleep‐related symptoms also illustrates how contextual factors, such as remote work, can indirectly influence symptom expression, highlighting the importance of tailoring interventions to the broader social environment.

### 4.1. Limitations and Future Directions

A few limitations of this study should be noted. First, this study only estimated networks for participants who had completed all three timepoints. This ensured a more cohesive comparison between networks, but also meant that several participants were excluded from the analyses, and significant group differences between included and excluded participants in baseline characteristics indicated that the results of this study may have limited generalizability since relatively older, highly educated and less depressed participants have been included. As well, this study provides novel information regarding the network structure and connectivity of anxiety and depression symptoms, however inferences drawn from the results of network analysis studies should carefully acknowledge that results do not imply causal association.

An additional limitation of this study is that the BAI and the QIDS items were combined into a single network. This approach risks generating artificial “communities” that mirror the original questionnaire structure (i.e., BAI vs. QIDS) rather than reflecting true symptom clustering. In addition, several BAI items, particularly somatic panic‐related items (e.g., trembling and shakiness), may be highly redundant, potentially inflating network connectivity. Redundancy diagnostics (e.g., the goldbricker procedure) and community detection approaches could be used in future work to address this issue. Nevertheless, we chose to retain all items as originally assessed to maximize clinical applicability and comparability with existing research, where BAI and QIDS are commonly used in their full form. This decision prioritizes interpretability for clinicians while acknowledging potential psychometric limitations.

Given that previous research has indicated that gender may also play a role in the mental health impact of COVID‐19, future studies could compare networks of female and male cohorts and how their symptoms changed and differed across the pandemic [35]. Future studies should also continue to apply longitudinal network methods with harmonized symptom datasets, enabling cumulative knowledge of symptom trajectories and identifying intervention targets at optimal timepoints.

## 5. Conclusions

While it is important to interpret the results of network analyses with caution, these findings provide novel evidence that the COVID‐19 pandemic reshaped the interconnections among anxiety and depressive symptoms in individuals with pre‐existing mental disorders. Compared to pre‐pandemic, symptom networks during the pandemic were less densely connected, suggesting weakened mutual reinforcement among symptoms under conditions of external stress. Importantly, we found a shift in centrality from acute fear‐related anxiety symptoms to appraisal‐based anxiety symptoms, alongside a reduction in the prominence of sleep‐related symptoms and suicidal ideation.

These findings underscore that the organization of psychopathology is not static but dynamically shaped by external societal stressors. By applying longitudinal network analysis to this dataset, we demonstrate the capacity to detect subtle shifts in symptom architecture that remain hidden when only sum scores are considered. This approach not only advances our theoretical understanding of mental health under crisis conditions but also provides a clinically actionable framework: interventions can be tailored to target the most central symptoms as they emerge in response to shifting contexts. In highlighting both the vulnerability and adaptability of symptom networks, our study emphasizes the importance of flexible, symptom‐level monitoring to guide prevention and treatment strategies in real time during future large‐scale crises.

## Author Contributions

**Sophie J. Blackmore:** conceptualization, methodology, formal analysis, data curation, writing – original draft, writing – review and editing. **Marit Sijbrandij and Brenda W. J. H. Penninx:** conceptualization, funding acquisition, writing – review and editing, supervision.**Jose Luis Ayuso-Mateos, Richard Bryant, Matteo Monzio Compagnoni, Katalin Gémes, Erik J. Giltay, Almar A. L. Kok, Vincent Lorant, Roberto Mediavilla, Josep Maria Haro, Raffael Kalisch, Maria Melchior, Ellenor Mittendorfer-Rutz, Pablo Nicaise, Papoula Petri-Romão, Judith van der Waerden, Henrik Walter, and Anke B. Witteveen:** conceptualization, funding acquisition, writing – review and editing.

## Funding

This study was funded by the Dutch Research Council (Grant 440.20.009). The infrastructure for the NESDA study is funded through the Geestkracht Programme of the Netherlands Organisation for Health Research and Development (Grant 10‐000‐1002) and financial contributions by participating universities and mental health‐care organizations (VU University Medical Center, Geestelijke gezondheidszorg (GGZ) inGeest, Leiden University Medical Center, Leiden University, GGZ Rivierduinen, University Medical Center Groningen, University of Groningen, Lentis, GGZ Friesland, GGZ Drenthe, Rob Giel Onderzoekscentrum). The infrastructure for the NESDO study is funded through the Fonds NutsOhra (Project 0701‐065), Stichting tot Steun VCVGZ, NARSAD The Brain and Behaviour Research Fund (Grant 41080), and by participating universities and mental health‐care organizations (VU University Medical Center, Leiden University Medical Center, University Medical Center Groningen, University Medical Center St Radboud, GGZ inGeest, GGNet, GGZ Nijmegen, GGZ Rivierduinen, Lentis, and Parnassia). The RESPOND Project is funded under Horizon 2020—the Framework Programme for Research and Innovation (2014−2020).

## Disclosure

The content of this article reflects only the authors’ views, and the European Community is not liable for any use that may be made of the information contained herein.

## Conflicts of Interest

The authors declare no conflicts of interest.

## Supporting Information

Additional supporting information can be found online in the Supporting Information section.

## Supporting information


**Supporting Information 1** Appendix E. Sensitivity analysis of network estimation across EBIC *γ* values. Figure E1. Sensitivity analysis of T0 network across *γ* values. Figure E2. Sensitivity analysis of T1 network across *γ* values. Figure E3. Sensitivity analysis of T2 network across *γ* values.

**Supporting Information 2** Appendix A. Demographic characteristics. Table A1. Table of demographic characteristics for the included study sample and excluded dropout participants.

**Supporting Information 3** Appendix B. Demographic characteristics comparisons. Table B1. Summary of chi‐squared results assessing differences in gender between included vs. excluded samples. Table B2. Summary table of *t*‐test results assessing differences in demographic characteristics between the included vs. excluded sample at baseline.

**Supporting Information 4** Appendix C. Plots of centrality indices. Figure C1. Centrality indices for the pre‐COVID timepoint. Figure C2. Centrality indices for the first COVID timepoint (April 2020). Figure C3. Centrality indices for the second COVID timepoint (January 2021).

**Supporting Information 5** Appendix D. Centrality stability plots. Figure D1. Stability of centrality indices, pre‐COVID timepoint. Figure D2. Stability of centrality indices, first COVID timepoint (April 2020). Figure D3. Stability of centrality indices, second COVID timepoint (January/February 2021).

**Supporting Information 6** Appendix F. Bootstrapped 95% confidence intervals. Table F1. Table of significant edges with bootstrapped 95% confidence intervals across the three timepoints.

## Data Availability

According to European law (General Data Protection Regulation), data containing potentially identifying or sensitive patients’ information are restricted. However, for academic researchers, data could be available upon request via the NESDA and NESDO (nesda@amsterdamumc.nl) data access committees.
