# Supplementary material for: Anxiety and Depressive Symptoms Before and During the COVID‐19 Pandemic: A Longitudinal Network Analysis
Source: Depress Anxiety. 2026 Mar 6;2026:9620883. doi: 10.1155/da/9620883 (PMC12965898; doi:10.1155/da/9620883)
Supplement: Supplementary file 1 — Supporting Information 1 Appendix E. Sensitivity analysis of network estimation across EBIC γ values. Figure E1. Sensitivity analysis of T0 network across γ values. Figure E2. Sensitivity analysis of T1 network across γ values. Figure E3. Sensitivity analysis of T2 network across γ values. [file DA-2026-9620883-s006.docx]

**Appendix E**

Sensitivity Analysis of Network Estimation Across EBIC γ Values

**Figure E1.**

*Sensitivity analysis of T0 network across γ values.*

**
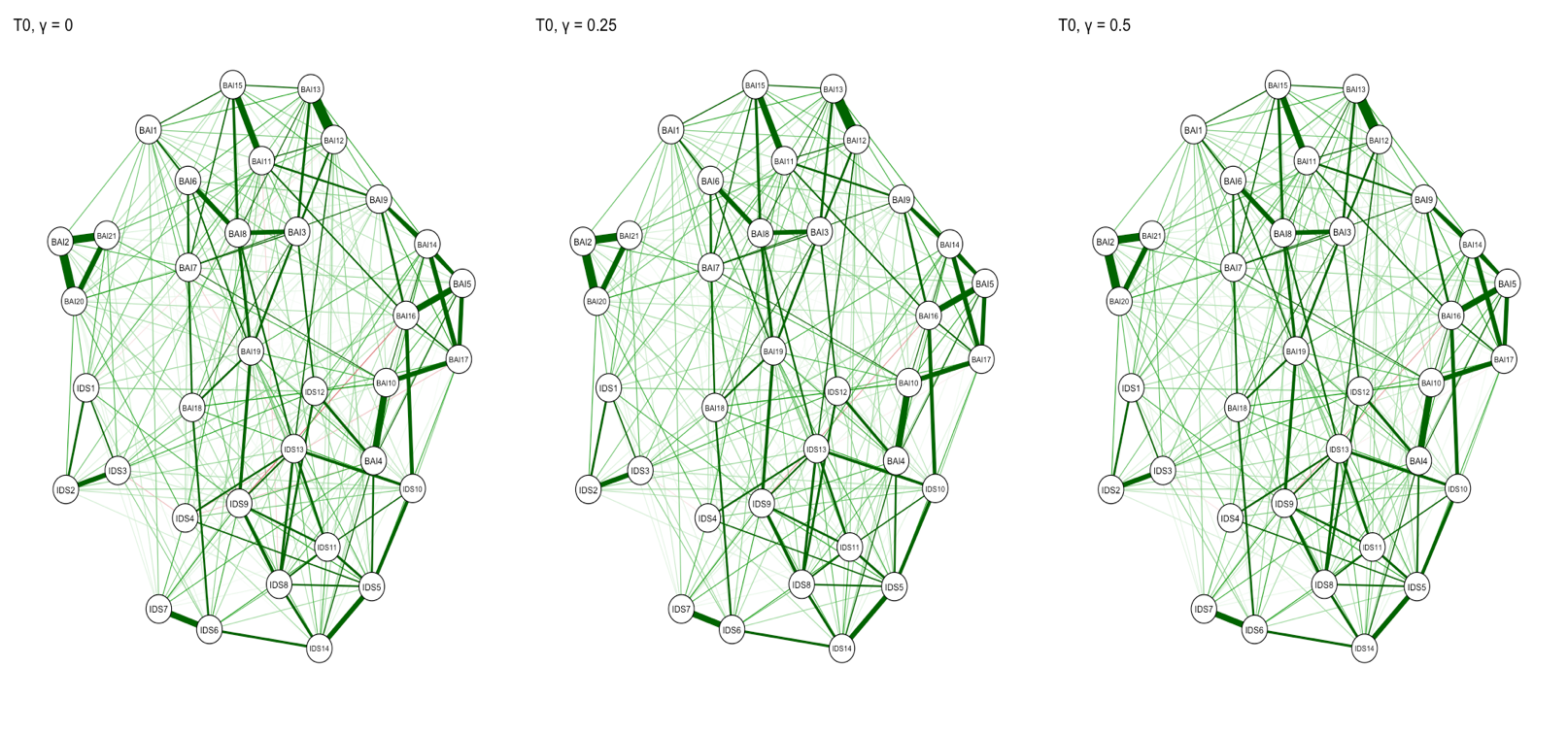
**

**Figure E2.**

*Sensitivity analysis of T1 network across γ values.*

**
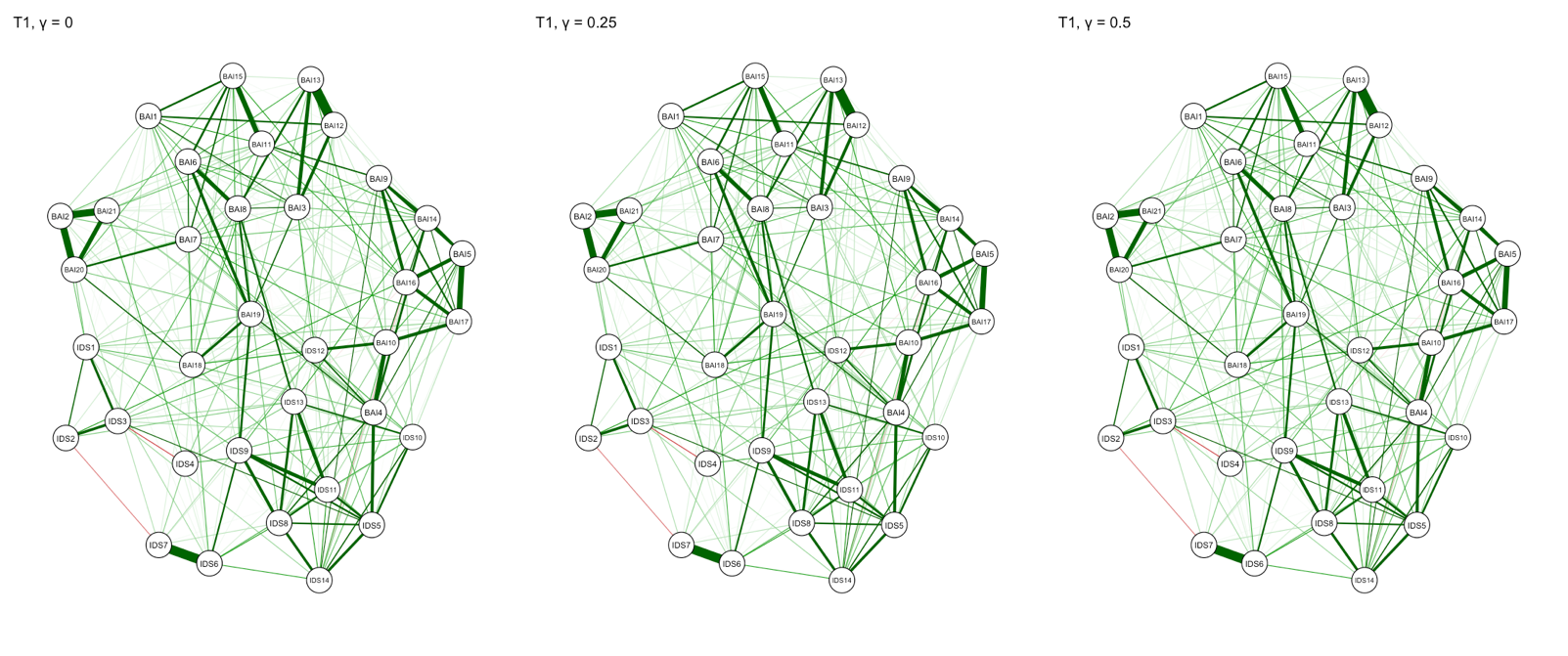
**

**Figure E3.**

*Sensitivity analysis of T2 network across γ values.*

*
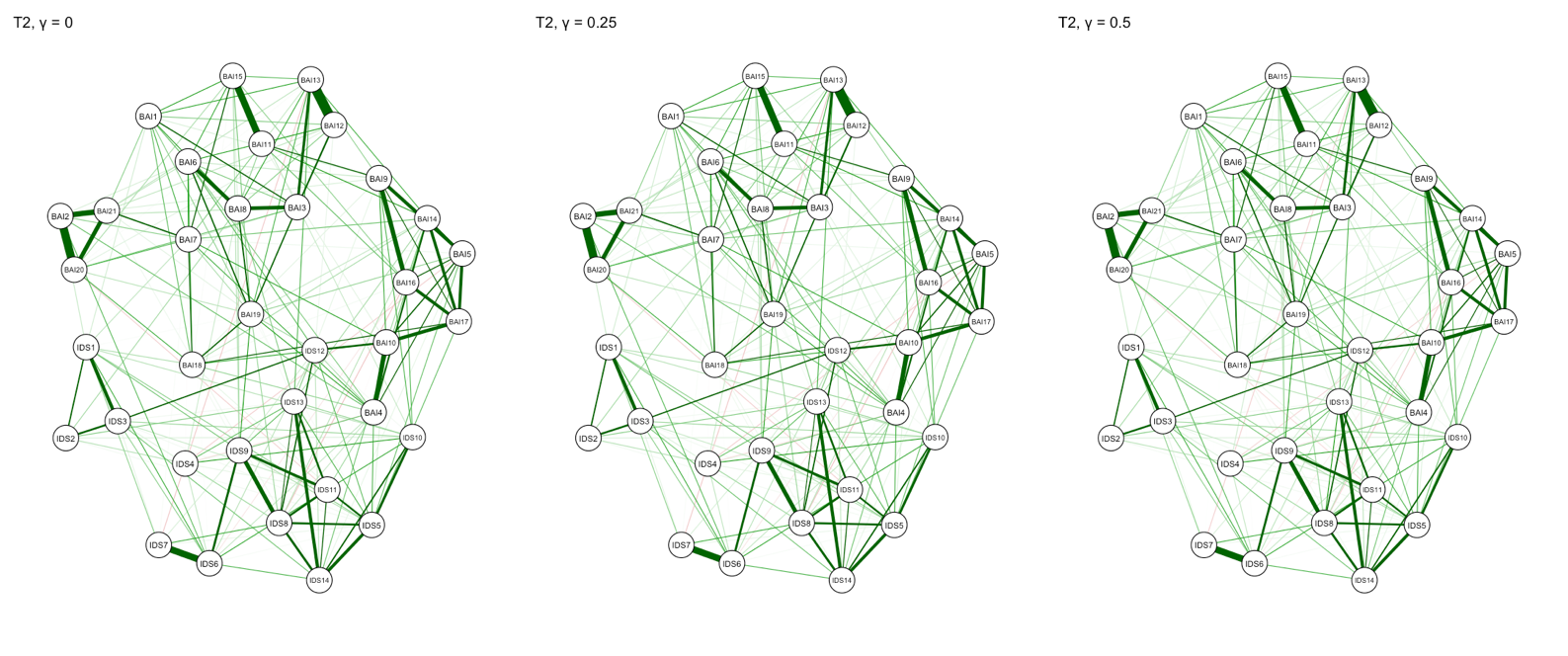
*

***Note:*** *Networks were re-estimated at each γ level following non-paranormal transformation. Visual inspection shows highly similar structures across specifications, with edge-weight correlations of 1.0 across all comparisons, indicating that network topology and connectivity patterns were robust to variation in the γ hyperparameter.*
