# Supplementary material for: Anxiety and Depressive Symptoms Before and During the COVID‐19 Pandemic: A Longitudinal Network Analysis
Source: Depress Anxiety. 2026 Mar 6;2026:9620883. doi: 10.1155/da/9620883 (PMC12965898; doi:10.1155/da/9620883)
Supplement: Supplementary file 2 — Supporting Information 2 Appendix A. Demographic characteristics. Table A1. Table of demographic characteristics for the included study sample and excluded dropout participants. [file DA-2026-9620883-s004.docx]

**Appendix A**

Demographic Characteristics

***Table A1.***

*Table of demographic characteristics for the included study sample and excluded dropout participants.*

| N (%) | | | |
| --- | --- | --- | --- |
|  | **Included subsample** | **Baseline Sample (Excluded participants)** | |
| **Number of participants** | 675 | | 1693 |
| **Gender**  *Male*  *Female* | 232 (34.4)  443 (65.6) | | 569 (33.6)  1124 (66.4) |
| Mean (SD) | | | |
| **Age at baseline timepoint** | 54.93 (12.46) | | 53.51 (15.71) |
| **Education level attained (years)** | 13.1 (3.18) | | 12.7 (3.47) |
| **BAI score**  *Pre-COVID T0*  *COVID T1*  *COVID T2*  **Total QIDS score**  *Pre-COVID T0*  *COVID T1*  *COVID T2* | 28.29 (8.38)  27.91 (7.9)  27.69 (8.07)  21.91 (6.55)  22.82 (6.52)  22.37 (6.73) | | 28.96 (8.54)  28.9 (9.07)  28.17 (8.80)  22.54 (6.83)  23.34 (6.96)  22.66 (6.86) |
