# Supplementary material for: Anxiety and Depressive Symptoms Before and During the COVID‐19 Pandemic: A Longitudinal Network Analysis
Source: Depress Anxiety. 2026 Mar 6;2026:9620883. doi: 10.1155/da/9620883 (PMC12965898; doi:10.1155/da/9620883)
Supplement: Supplementary file 3 — Supporting Information 3 Appendix B. Demographic characteristics comparisons. Table B1. Summary of chi‐squared results assessing differences in gender between included vs. excluded samples. Table B2. Summary table of t‐test results assessing differences in demographic characteristics between the included vs. excluded sample at baseline. [file DA-2026-9620883-s003.docx]

**Appendix B**

Demographic Characteristics Comparisons

**Table B1.**

*Summary of chi-squared results assessing differences in gender between included vs excluded samples.*

|  | **Chi-square** | **df** | **p-value** |
| --- | --- | --- | --- |
| **Gender*participation** | **.142** | **1** | **.706** |

**Table B2.**

*Summary table of t-test results assessing differences in demographic characteristics between the included vs excluded sample at baseline.*

|  | **Included sample (n = 675)** | **Excluded sample (n = 1693)** |  |  |  |
| --- | --- | --- | --- | --- | --- |
|  | **Mean (SD)** | **Mean (SD)** | **t(df)** | **p** | **Cohen’s d** |
| **Age at baseline** | 54.93 (12.46) | 53.51 (15.71) | -2.319(1547.01) | .021* | -.096 |
| **Education level attained (years)** | 12.7 (3.47) | 13.1 (3.18) | -2.56(2365) | .01* | -.117 |
| **BAI Score at baseline** | 28.29 (8.38) | 28.96 (8.54) | 1.668(2166) | .095 | .078 |
| **QIDS Score at baseline** | 21.91 (6.55) | 22.54 (6.83) | 2.004(2212) | .045* | .093 |

*Note: * = significant at p < .05.*
