# Supplementary material for: Anxiety and Depressive Symptoms Before and During the COVID‐19 Pandemic: A Longitudinal Network Analysis
Source: Depress Anxiety. 2026 Mar 6;2026:9620883. doi: 10.1155/da/9620883 (PMC12965898; doi:10.1155/da/9620883)
Supplement: Supplementary file 4 — Supporting Information 4 Appendix C. Plots of centrality indices. Figure C1. Centrality indices for the pre‐COVID timepoint. Figure C2. Centrality indices for the first COVID timepoint (April 2020). Figure C3. Centrality indices for the second COVID timepoint (January 2021). [file DA-2026-9620883-s002.docx]

**Appendix C**

Plots of Centrality Indices

**Figure C1.**

*Centrality indices for the pre-COVID timepoint.*

*
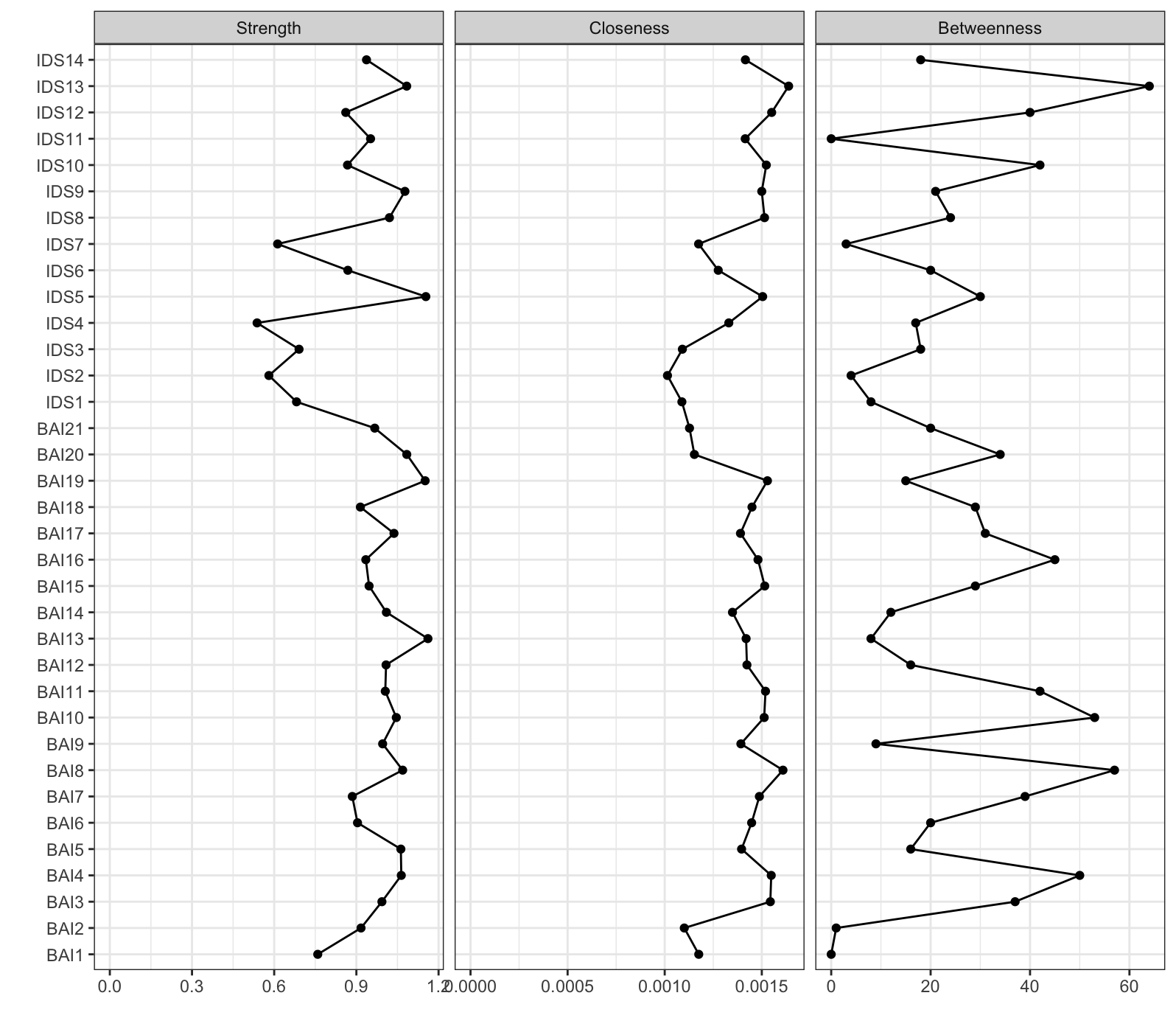
*

***Note****: Higher numbers indicate an item is more central to the network. Values on the x-axis are standardized z-scores.*

**Figure C2.**

*Centrality indices for the first COVID timepoint (April 2020).*

*
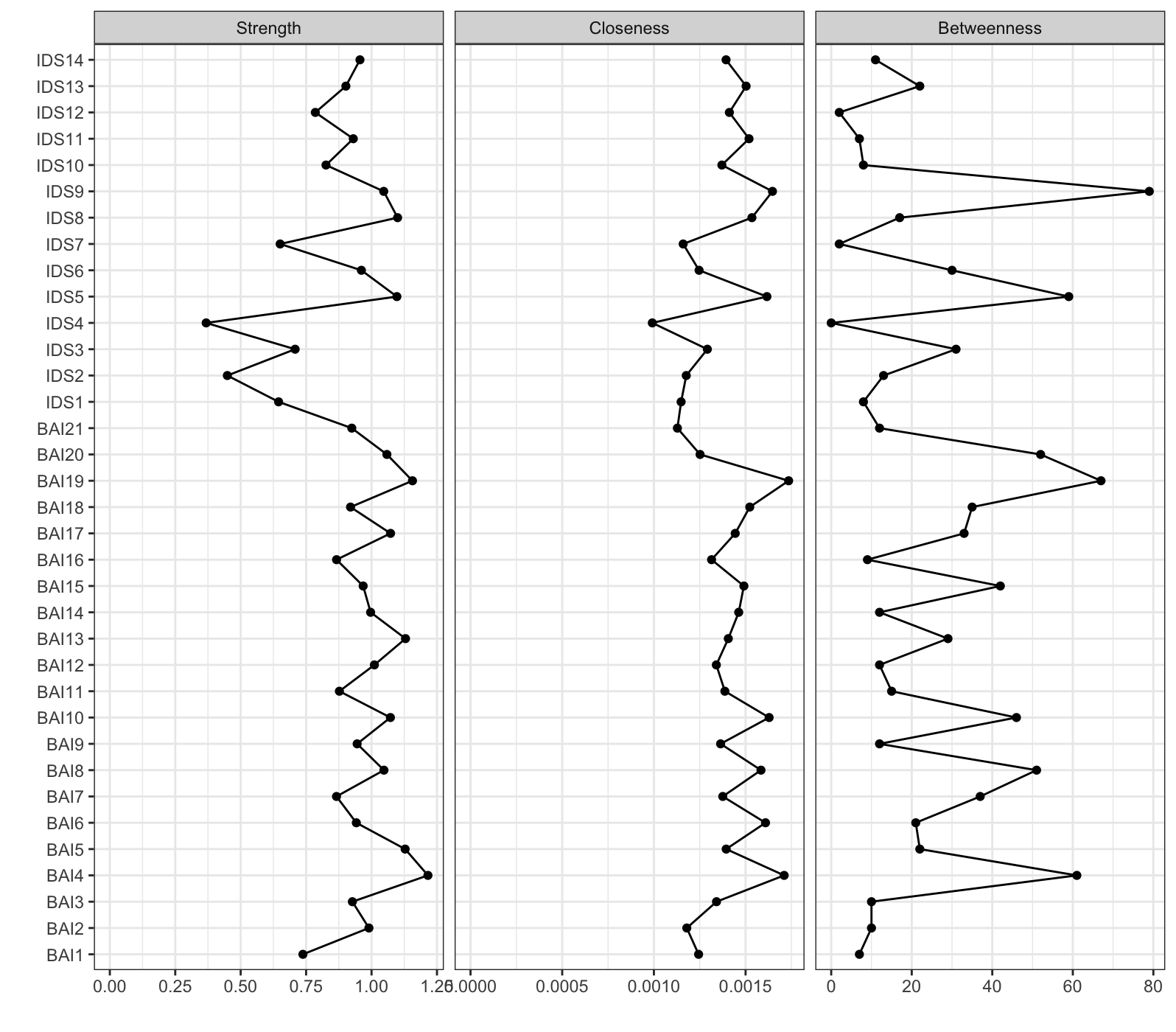
*

***Note****: Higher numbers indicate an item is more central to the network. Values on the x-axis are standardized z-scores.*

**Figure C3.**

*Centrality indices for the second COVID timepoint (January 2021).*

*
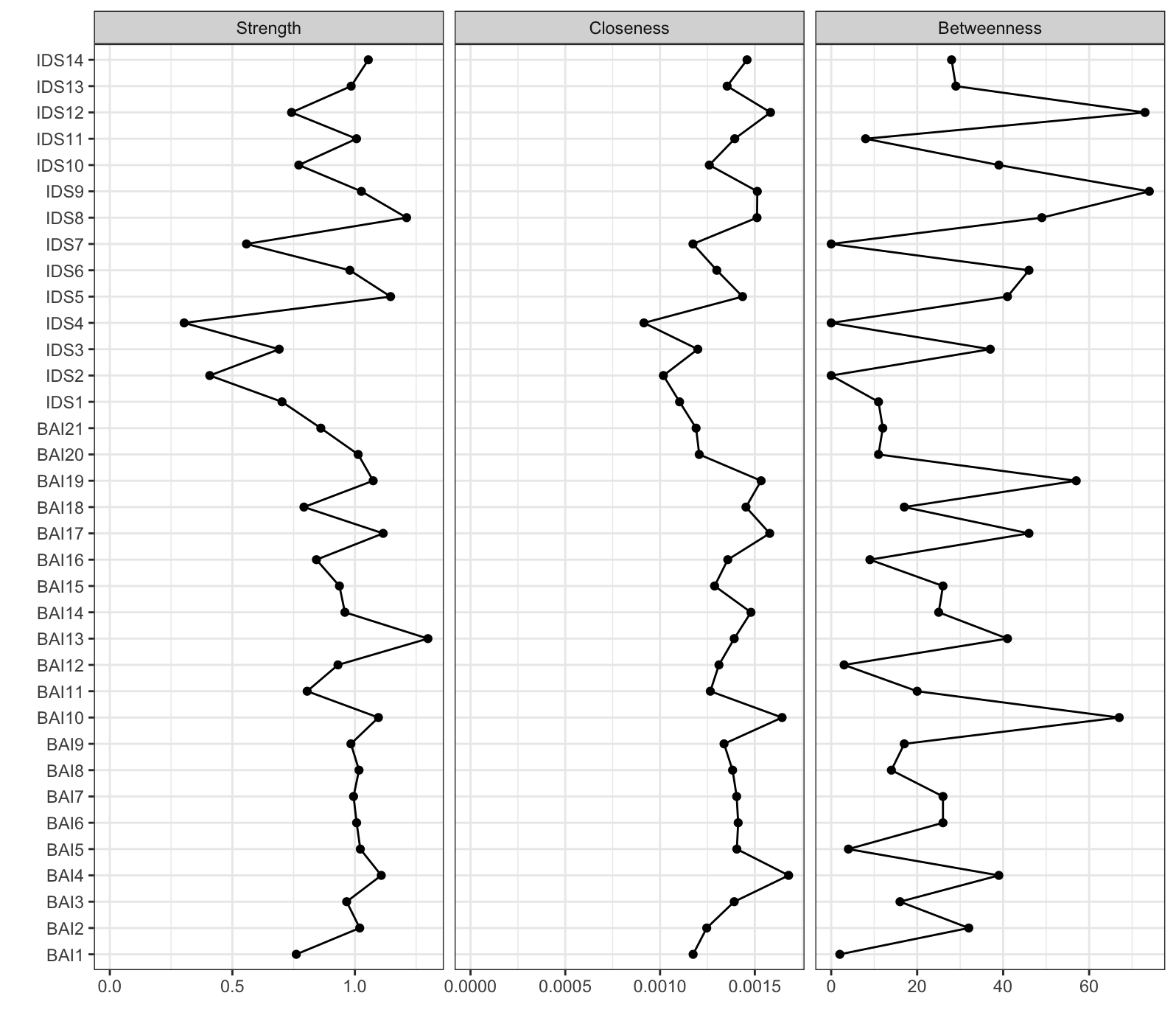
*

***Note****: Higher numbers indicate an item is more central to the network. Values on the x-axis are standardized z-scores.*
