# Supplementary material for: Anxiety and Depressive Symptoms Before and During the COVID‐19 Pandemic: A Longitudinal Network Analysis
Source: Depress Anxiety. 2026 Mar 6;2026:9620883. doi: 10.1155/da/9620883 (PMC12965898; doi:10.1155/da/9620883)
Supplement: Supplementary file 5 — Supporting Information 5 Appendix D. Centrality stability plots. Figure D1. Stability of centrality indices, pre‐COVID timepoint. Figure D2. Stability of centrality indices, first COVID timepoint (April 2020). Figure D3. Stability of centrality indices, second COVID timepoint (January/February 2021). [file DA-2026-9620883-s001.docx]

**Appendix D**

Centrality Stability Plots

**Figure D1.**

*Stability of centrality indices, pre-COVID timepoint.*

*
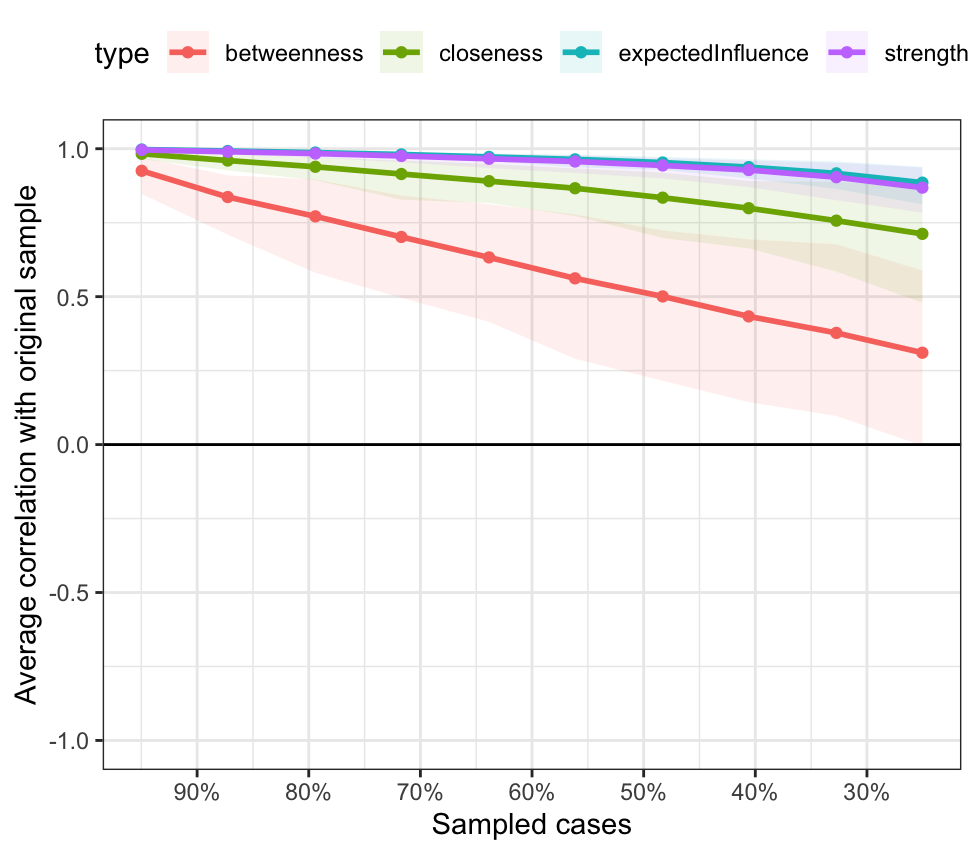
*

***Note****: Demonstrates the average correlation between bootstrap centrality indices of networks sampled with node-dropping and baseline network. Average correlation values less than 0.5 at 30% samples cases indicates a weak to moderate correlation between the original sample’s centrality measure and the bootstrapped samples’ values.*

**Figure D2.**

*Stability of centrality indices, first COVID timepoint (April 2020).*

*
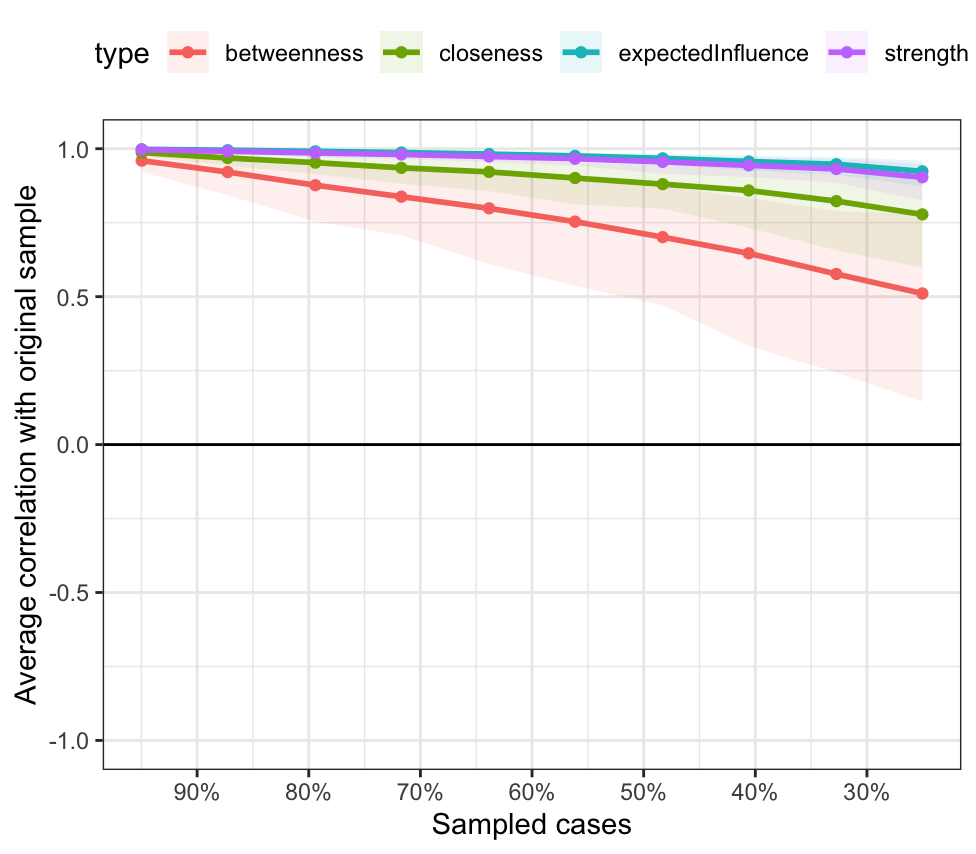
*

***Note****: Demonstrates the average correlation between bootstrap centrality indices of networks sampled with node-dropping and the first COVID timepoint network. Average correlation values less than 0.5 at 30% samples cases indicates a weak to moderate correlation between the original sample’s centrality measure and the bootstrapped samples’ values.*

**Figure D3.**

*Stability of centrality indices, second COVID timepoint (January/February 2021).*

*
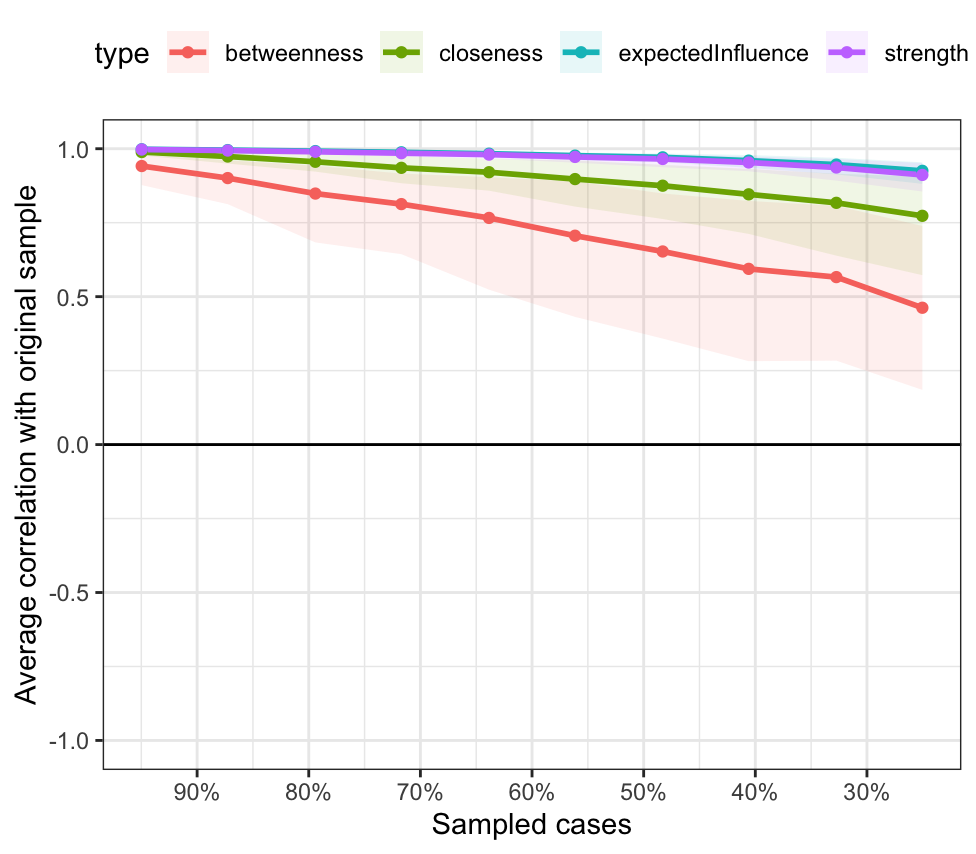
*

***Note****: Demonstrates the average correlation between bootstrap centrality indices of networks sampled with node-dropping and the second COVID timepoint network. Average correlation values less than 0.5 at 30% samples cases indicates a weak to moderate correlation between the original sample’s centrality measure and the bootstrapped samples’ values.*
