# Supplementary material for: Anxiety and Depressive Symptoms Before and During the COVID‐19 Pandemic: A Longitudinal Network Analysis
Source: Depress Anxiety. 2026 Mar 6;2026:9620883. doi: 10.1155/da/9620883 (PMC12965898; doi:10.1155/da/9620883)
Supplement: Supplementary file 6 — Supporting Information 6 Appendix F. Bootstrapped 95% confidence intervals. Table F1. Table of significant edges with bootstrapped 95% confidence intervals across the three timepoints. [file DA-2026-9620883-s005.docx]

# Appendix F

# Bootstrapped 95% Confidence Intervals

# *Table F1.*

# *Table of significant edges with bootstrapped 95% confidence intervals across the three timepoints.*

| **Timepoint** | **Edge** | **Mean Weight** | **95% CI Lower** | **95% CI Upper** |
| --- | --- | --- | --- | --- |
| T0 | BAI1–BAI6 | 0.096 | 0.021 | 0.173 |
| T0 | BAI10–BAI14 | 0.081 | 0.011 | 0.151 |
| T0 | BAI10–BAI17 | 0.213 | 0.137 | 0.290 |
| T0 | BAI10–IDS12 | 0.070 | 0.005 | 0.139 |
| T0 | BAI11–BAI15 | 0.281 | 0.187 | 0.376 |
| T0 | BAI12–BAI13 | 0.422 | 0.319 | 0.519 |
| T0 | BAI12–IDS13 | 0.076 | 0.002 | 0.159 |
| T0 | BAI14–BAI17 | 0.218 | 0.131 | 0.303 |
| T0 | BAI16–BAI17 | 0.096 | 0.023 | 0.173 |
| T0 | BAI16–IDS10 | 0.175 | 0.084 | 0.266 |
| T0 | BAI18–BAI19 | 0.109 | 0.036 | 0.184 |
| T0 | BAI18–IDS6 | 0.108 | 0.031 | 0.183 |
| T0 | BAI19–IDS9 | 0.157 | 0.087 | 0.229 |
| T0 | BAI2–BAI20 | 0.378 | 0.299 | 0.452 |
| T0 | BAI2–BAI21 | 0.325 | 0.252 | 0.400 |
| T0 | BAI20–BAI21 | 0.246 | 0.161 | 0.330 |
| T0 | BAI3–BAI12 | 0.118 | 0.024 | 0.213 |
| T0 | BAI3–BAI13 | 0.144 | 0.045 | 0.244 |
| T0 | BAI3–BAI19 | 0.126 | 0.047 | 0.202 |
| T0 | BAI3–BAI8 | 0.198 | 0.103 | 0.290 |
| T0 | BAI3–IDS12 | 0.096 | 0.019 | 0.175 |
| T0 | BAI4–BAI10 | 0.300 | 0.223 | 0.379 |
| T0 | BAI4–IDS12 | 0.137 | 0.070 | 0.205 |
| T0 | BAI4–IDS14 | 0.079 | 0.003 | 0.155 |
| T0 | BAI4–IDS5 | 0.108 | 0.042 | 0.177 |
| T0 | BAI5–BAI14 | 0.146 | 0.059 | 0.229 |
| T0 | BAI5–BAI16 | 0.262 | 0.178 | 0.349 |
| T0 | BAI5–BAI17 | 0.204 | 0.116 | 0.293 |
| T0 | BAI5–BAI9 | 0.142 | 0.060 | 0.223 |
| T0 | BAI6–BAI19 | 0.090 | 0.017 | 0.160 |
| T0 | BAI6–BAI7 | 0.114 | 0.033 | 0.198 |
| T0 | BAI6–BAI8 | 0.213 | 0.131 | 0.290 |
| T0 | BAI7–BAI15 | 0.086 | 0.001 | 0.168 |
| T0 | BAI7–BAI18 | 0.105 | 0.031 | 0.184 |
| T0 | BAI8–BAI15 | 0.134 | 0.043 | 0.226 |
| T0 | BAI8–BAI19 | 0.149 | 0.069 | 0.231 |
| T0 | BAI8–IDS13 | 0.098 | 0.021 | 0.185 |
| T0 | BAI9–BAI14 | 0.154 | 0.071 | 0.238 |
| T0 | BAI9–BAI16 | 0.132 | 0.028 | 0.242 |
| T0 | IDS1–IDS2 | 0.120 | 0.049 | 0.186 |
| T0 | IDS1–IDS3 | 0.088 | 0.009 | 0.166 |
| T0 | IDS10–IDS13 | 0.132 | 0.051 | 0.218 |
| T0 | IDS11–IDS13 | 0.135 | 0.043 | 0.234 |
| T0 | IDS2–IDS3 | 0.212 | 0.140 | 0.280 |
| T0 | IDS4–IDS13 | 0.095 | 0.016 | 0.173 |
| T0 | IDS4–IDS5 | 0.076 | 0.012 | 0.141 |
| T0 | IDS5–IDS10 | 0.161 | 0.091 | 0.233 |
| T0 | IDS5–IDS11 | 0.129 | 0.054 | 0.205 |
| T0 | IDS5–IDS14 | 0.222 | 0.140 | 0.299 |
| T0 | IDS5–IDS8 | 0.102 | 0.023 | 0.182 |
| T0 | IDS6–IDS14 | 0.123 | 0.039 | 0.209 |
| T0 | IDS6–IDS7 | 0.253 | 0.172 | 0.331 |
| T0 | IDS8–IDS11 | 0.103 | 0.024 | 0.184 |
| T0 | IDS8–IDS12 | 0.118 | 0.038 | 0.196 |
| T0 | IDS8–IDS13 | 0.135 | 0.051 | 0.216 |
| T0 | IDS8–IDS14 | 0.137 | 0.052 | 0.217 |
| T0 | IDS8–IDS9 | 0.164 | 0.090 | 0.239 |
| T0 | IDS9–IDS11 | 0.115 | 0.045 | 0.191 |
| T0 | IDS9–IDS13 | 0.098 | 0.020 | 0.172 |
| T1 | BAI1–BAI12 | 0.100 | 0.023 | 0.181 |
| T1 | BAI1–BAI15 | 0.120 | 0.034 | 0.208 |
| T1 | BAI1–BAI3 | 0.079 | 0.003 | 0.161 |
| T1 | BAI10–BAI14 | 0.087 | 0.007 | 0.163 |
| T1 | BAI10–BAI17 | 0.189 | 0.118 | 0.258 |
| T1 | BAI10–IDS12 | 0.163 | 0.100 | 0.222 |
| T1 | BAI11–BAI15 | 0.267 | 0.172 | 0.359 |
| T1 | BAI12–BAI13 | 0.479 | 0.378 | 0.571 |
| T1 | BAI14–BAI17 | 0.080 | 0.001 | 0.155 |
| T1 | BAI16–BAI17 | 0.194 | 0.123 | 0.265 |
| T1 | BAI18–BAI19 | 0.148 | 0.078 | 0.218 |
| T1 | BAI18–BAI20 | 0.089 | 0.019 | 0.162 |
| T1 | BAI19–IDS9 | 0.124 | 0.054 | 0.193 |
| T1 | BAI2–BAI20 | 0.354 | 0.275 | 0.426 |
| T1 | BAI2–BAI21 | 0.360 | 0.276 | 0.441 |
| T1 | BAI20–BAI21 | 0.238 | 0.147 | 0.326 |
| T1 | BAI3–BAI12 | 0.177 | 0.086 | 0.271 |
| T1 | BAI3–BAI13 | 0.208 | 0.117 | 0.302 |
| T1 | BAI3–BAI19 | 0.090 | 0.018 | 0.163 |
| T1 | BAI4–BAI10 | 0.227 | 0.157 | 0.295 |
| T1 | BAI4–BAI14 | 0.130 | 0.063 | 0.199 |
| T1 | BAI4–BAI19 | 0.078 | 0.013 | 0.141 |
| T1 | BAI4–BAI5 | 0.084 | 0.016 | 0.144 |
| T1 | BAI4–IDS12 | 0.103 | 0.031 | 0.173 |
| T1 | BAI4–IDS5 | 0.178 | 0.107 | 0.244 |
| T1 | BAI4–IDS8 | 0.096 | 0.028 | 0.164 |
| T1 | BAI5–BAI14 | 0.131 | 0.046 | 0.212 |
| T1 | BAI5–BAI16 | 0.202 | 0.125 | 0.279 |
| T1 | BAI5–BAI17 | 0.299 | 0.225 | 0.373 |
| T1 | BAI5–BAI9 | 0.154 | 0.077 | 0.226 |
| T1 | BAI6–BAI15 | 0.128 | 0.053 | 0.205 |
| T1 | BAI6–BAI19 | 0.168 | 0.093 | 0.239 |
| T1 | BAI6–BAI8 | 0.211 | 0.128 | 0.291 |
| T1 | BAI7–BAI15 | 0.099 | 0.018 | 0.177 |
| T1 | BAI7–BAI20 | 0.119 | 0.043 | 0.193 |
| T1 | BAI8–BAI13 | 0.123 | 0.044 | 0.201 |
| T1 | BAI8–BAI19 | 0.124 | 0.050 | 0.198 |
| T1 | BAI8–IDS13 | 0.112 | 0.037 | 0.191 |
| T1 | BAI9–BAI11 | 0.097 | 0.003 | 0.199 |
| T1 | BAI9–BAI14 | 0.133 | 0.051 | 0.219 |
| T1 | BAI9–BAI16 | 0.178 | 0.090 | 0.271 |
| T1 | BAI9–BAI17 | 0.117 | 0.052 | 0.180 |
| T1 | IDS1–IDS2 | 0.085 | 0.011 | 0.156 |
| T1 | IDS1–IDS3 | 0.141 | 0.059 | 0.219 |
| T1 | IDS10–IDS13 | 0.100 | 0.007 | 0.195 |
| T1 | IDS10–IDS14 | 0.089 | 0.007 | 0.173 |
| T1 | IDS11–IDS13 | 0.192 | 0.102 | 0.279 |
| T1 | IDS2–IDS3 | 0.157 | 0.082 | 0.226 |
| T1 | IDS3–IDS5 | 0.070 | 0.001 | 0.136 |
| T1 | IDS5–IDS10 | 0.125 | 0.059 | 0.193 |
| T1 | IDS5–IDS11 | 0.138 | 0.062 | 0.217 |
| T1 | IDS5–IDS14 | 0.162 | 0.082 | 0.244 |
| T1 | IDS5–IDS8 | 0.114 | 0.035 | 0.197 |
| T1 | IDS5–IDS9 | 0.119 | 0.045 | 0.194 |
| T1 | IDS6–IDS7 | 0.459 | 0.394 | 0.517 |
| T1 | IDS6–IDS9 | 0.110 | 0.042 | 0.176 |
| T1 | IDS8–IDS11 | 0.121 | 0.039 | 0.206 |
| T1 | IDS8–IDS13 | 0.150 | 0.075 | 0.219 |
| T1 | IDS8–IDS14 | 0.157 | 0.071 | 0.242 |
| T1 | IDS8–IDS9 | 0.177 | 0.094 | 0.253 |
| T1 | IDS9–IDS11 | 0.199 | 0.118 | 0.281 |
| T2 | BAI1–BAI13 | 0.086 | 0.009 | 0.169 |
| T2 | BAI1–BAI3 | 0.115 | 0.024 | 0.205 |
| T2 | BAI10–BAI17 | 0.223 | 0.151 | 0.293 |
| T2 | BAI10–BAI18 | 0.075 | 0.004 | 0.144 |
| T2 | BAI10–IDS12 | 0.144 | 0.076 | 0.219 |
| T2 | BAI11–BAI14 | 0.081 | 0.011 | 0.151 |
| T2 | BAI11–BAI15 | 0.374 | 0.293 | 0.458 |
| T2 | BAI12–BAI13 | 0.517 | 0.429 | 0.601 |
| T2 | BAI13–BAI14 | 0.071 | 0.014 | 0.128 |
| T2 | BAI13–BAI19 | 0.071 | 0.001 | 0.139 |
| T2 | BAI14–BAI17 | 0.195 | 0.121 | 0.267 |
| T2 | BAI16–BAI17 | 0.204 | 0.131 | 0.283 |
| T2 | BAI17–BAI18 | 0.095 | 0.025 | 0.165 |
| T2 | BAI17–IDS5 | 0.058 | 0.002 | 0.116 |
| T2 | BAI18–BAI19 | 0.109 | 0.034 | 0.187 |
| T2 | BAI18–BAI20 | 0.070 | 0.005 | 0.134 |
| T2 | BAI19–IDS9 | 0.085 | 0.023 | 0.148 |
| T2 | BAI2–BAI20 | 0.448 | 0.374 | 0.523 |
| T2 | BAI2–BAI21 | 0.313 | 0.231 | 0.392 |
| T2 | BAI2–BAI4 | 0.053 | 0.005 | 0.102 |
| T2 | BAI2–IDS6 | 0.058 | 0.005 | 0.108 |
| T2 | BAI20–BAI21 | 0.260 | 0.179 | 0.345 |
| T2 | BAI3–BAI12 | 0.140 | 0.045 | 0.236 |
| T2 | BAI3–BAI13 | 0.185 | 0.095 | 0.272 |
| T2 | BAI3–BAI19 | 0.122 | 0.043 | 0.199 |
| T2 | BAI3–BAI8 | 0.231 | 0.143 | 0.316 |
| T2 | BAI4–BAI10 | 0.274 | 0.201 | 0.344 |
| T2 | BAI4–BAI14 | 0.135 | 0.070 | 0.202 |
| T2 | BAI4–BAI19 | 0.085 | 0.016 | 0.153 |
| T2 | BAI4–BAI5 | 0.103 | 0.035 | 0.174 |
| T2 | BAI4–BAI7 | 0.075 | 0.007 | 0.144 |
| T2 | BAI4–IDS5 | 0.073 | 0.006 | 0.134 |
| T2 | BAI5–BAI10 | 0.115 | 0.044 | 0.187 |
| T2 | BAI5–BAI14 | 0.122 | 0.046 | 0.195 |
| T2 | BAI5–BAI16 | 0.102 | 0.023 | 0.179 |
| T2 | BAI5–BAI17 | 0.207 | 0.134 | 0.285 |
| T2 | BAI5–BAI9 | 0.215 | 0.142 | 0.290 |
| T2 | BAI6–BAI12 | 0.078 | 0.004 | 0.150 |
| T2 | BAI6–BAI15 | 0.077 | 0.003 | 0.152 |
| T2 | BAI6–BAI19 | 0.122 | 0.048 | 0.197 |
| T2 | BAI6–BAI7 | 0.088 | 0.015 | 0.160 |
| T2 | BAI6–BAI8 | 0.229 | 0.152 | 0.307 |
| T2 | BAI7–BAI10 | 0.080 | 0.012 | 0.148 |
| T2 | BAI7–BAI15 | 0.107 | 0.028 | 0.188 |
| T2 | BAI7–BAI18 | 0.105 | 0.029 | 0.180 |
| T2 | BAI7–BAI21 | 0.118 | 0.042 | 0.191 |
| T2 | BAI8–BAI13 | 0.083 | 0.008 | 0.163 |
| T2 | BAI8–BAI19 | 0.124 | 0.053 | 0.197 |
| T2 | BAI9–BAI10 | 0.077 | 0.004 | 0.143 |
| T2 | BAI9–BAI11 | 0.095 | 0.006 | 0.184 |
| T2 | BAI9–BAI16 | 0.269 | 0.177 | 0.359 |
| T2 | BAI9–BAI17 | 0.097 | 0.023 | 0.171 |
| T2 | IDS1–IDS2 | 0.118 | 0.043 | 0.185 |
| T2 | IDS1–IDS3 | 0.213 | 0.135 | 0.288 |
| T2 | IDS1–IDS6 | 0.078 | 0.009 | 0.149 |
| T2 | IDS10–IDS14 | 0.118 | 0.035 | 0.200 |
| T2 | IDS11–IDS13 | 0.149 | 0.064 | 0.235 |
| T2 | IDS11–IDS14 | 0.107 | 0.023 | 0.192 |
| T2 | IDS13–IDS14 | 0.208 | 0.120 | 0.300 |
| T2 | IDS2–IDS3 | 0.141 | 0.067 | 0.212 |
| T2 | IDS3–IDS12 | 0.102 | 0.025 | 0.176 |
| T2 | IDS4–IDS10 | 0.083 | 0.005 | 0.159 |
| T2 | IDS5–IDS10 | 0.167 | 0.094 | 0.242 |
| T2 | IDS5–IDS11 | 0.147 | 0.070 | 0.227 |
| T2 | IDS5–IDS14 | 0.205 | 0.128 | 0.280 |
| T2 | IDS5–IDS8 | 0.153 | 0.083 | 0.228 |
| T2 | IDS6–IDS11 | 0.082 | 0.013 | 0.152 |
| T2 | IDS6–IDS7 | 0.368 | 0.303 | 0.434 |
| T2 | IDS6–IDS9 | 0.159 | 0.094 | 0.229 |
| T2 | IDS7–IDS8 | 0.069 | 0.010 | 0.130 |
| T2 | IDS8–IDS11 | 0.159 | 0.080 | 0.239 |
| T2 | IDS8–IDS12 | 0.113 | 0.035 | 0.190 |
| T2 | IDS8–IDS13 | 0.100 | 0.027 | 0.173 |
| T2 | IDS8–IDS14 | 0.165 | 0.088 | 0.239 |
| T2 | IDS8–IDS9 | 0.252 | 0.180 | 0.324 |
| T2 | IDS9–IDS11 | 0.181 | 0.102 | 0.261 |

Note. Listed edges are those whose bootstrapped 95% confidence intervals did not include zero, indicating statistically reliable associations. Mean values are estimated edge weights.
